# Methods and Models for Studying *Mycobacterium tuberculosis* in Respiratory Infections

**DOI:** 10.3390/ijms26010018

**Published:** 2024-12-24

**Authors:** Caterina Franco, Rita Rezzani

**Affiliations:** 1 Anatomy and Physiopathology Division, Department of Clinical and Experimental Sciences, University of Brescia, 25123 Brescia, Italy; 2 Division of Immunology, Transplantation, and Infectious Diseases, IRCCS San Raffaele Scientific Institute, 20132 Milan, Italy; 3 Italian Society for the Study of Orofacial Pain (Società Italiana Studio Dolore Orofacciale–SISDO), 25123 Brescia, Italy; 4 Interdepartmental University Center of Research “Adaption and Regeneration of Tissues and Organs (ARTO)”, University of Brescia, 25123 Brescia, Italy

**Keywords:** tuberculosis, *Mycobacterium tuberculosis*, in vivo and in vitro studies, animal models, 3D in vitro system, granuloma

## Abstract

Respiratory infections, including tuberculosis, constitute a major global health challenge. Tuberculosis (TB), caused by *Mycobacterium tuberculosis* (Mtb), remains one of the leading causes of mortality worldwide. The disease’s complexity is attributed to Mtb’s capacity to persist in latent states, evade host immune defenses, and develop resistance to antimicrobial treatments, posing significant challenges for diagnosis and therapy. Traditional models, such as animal studies and two-dimensional (2D) in vitro systems, often fail to accurately recapitulate human-specific immune processes, particularly the formation of granulomas—a defining feature of tubercular infection. These limitations underscore the need for more physiologically relevant models to study TB pathogenesis. Emerging three-dimensional (3D) in vitro systems, including organoids and lung-on-chip platforms, offer innovative approaches to mimic the structural and functional complexity of the human lung. These models enable the recreation of key aspects of the tubercular granulomas, such as cellular interactions, oxygen gradients, and nutrient limitations, thereby providing deeper insights into Mtb pathogenesis. This review aims to elucidate the advantages of 3D in vitro systems in bridging the translational gap between traditional experimental approaches and clinical applications. Particular emphasis is placed on their potential to address challenges related to genetic variability in both the host and pathogen, thereby advancing tubercular research and therapeutic development.

## 1. Introduction

The lung is a complex organ in which it is possible to find more than 50 cell types with different origins. These cells are distributed across different lung compartments, such as airways, blood and lymphatic vessels, connective tissue, nerves, the mesothelium, and the lung parenchyma. Moreover, they are exposed to various stimuli, highlighting the high complexity of lung organization [1].

Respiratory infections are prevalent worldwide and were already rising before the COVID-19 pandemic, leading to significant morbidity and mortality. They rank as the third-leading cause of premature death and are linked to many chronic respiratory conditions [2].

Additionally, recent studies have demonstrated the involvement of respiratory infections, both viral and bacterial, in the development and progression of lung cancer.

For this reason, understanding how respiratory infective diseases behave in models that mimic physiological lung conditions is essential for developing effective vaccines and treatments [3].

Among all the pulmonary infectious pathogens, *Mycobacterium tuberculosis* (Mtb), the bacteria responsible for tuberculosis (TB), is one of the two leading causes of infectious death worldwide [4].

TB is one of the oldest acquired infectious diseases, constituting a persistent threat to public health. Designated as a global emergency by the World Health Organization (WHO) in 1993, it has infected approximately 10.6 million individuals worldwide and caused 1.3 million deaths, making it one of the primary causes of mortality attributed to a single infectious agent. Additionally, an estimated one-fourth of the global population (~1.7 billion people) carries latent tuberculosis infection (LTBI) [5].

It is important to note that Mtb can adapt to the human host continuously, and this specific feature adds another layer of complexity to TB management alongside the emergence of drug-resistant strains.

Thus, developing new models to study Mtb infection, from the initial stages through granuloma formation, is essential for advancing our understanding of TB and improving treatment and prevention strategies. Current models, particularly animal and in vitro systems, provide limited insight due to significant differences between human and model immune responses, especially in granuloma formation, where human-specific immune processes play a critical role [6]. Granulomas, which are organized structures formed by immune cells to contain the infection, involve complex host–pathogen interactions, including a balance between immune containment and tissue damage, which is difficult to replicate outside of human systems [6]. Traditional models often do not capture this dynamic or support long-term studies that simulate the months or years over which TB progresses in humans [7]. New approaches are emerging, such as 3D in vitro culture models using human cells to closely approximate granuloma structures, as well as lung organoid and organ-on-chip models that mimic human lung architecture and physiological conditions [8]. Additionally, humanized animal models with human immune cells offer more accurate reflections of the immune response to TB and show promise in understanding granuloma dynamics and immune interactions [9]. Combined with advanced imaging and computational techniques, these models allow for the real-time visualization and analysis of granuloma development and can simulate various aspects of TB pathogenesis under controlled conditions [10].

Ultimately, developing new models will accelerate the discovery of effective therapies and vaccines, improve our understanding of TB progression, and enable the identification of novel therapeutic targets, all of which are critical to addressing the global TB epidemic. Furthermore, there is an urgent need for rapid and accurate diagnostic tools to control TB transmission and enhance treatment outcomes. The development of new physiologic three-dimensional (3D) models has the potential to significantly accelerate progress in this area, improving early detection and supporting more effective disease management.

This review aims to bring together current knowledge about the respiratory models used in tuberculosis research, highlighting where we stand today and paving the way for future studies. We will also take a closer look at animal models and the recently developed three-dimensional granuloma models, discussing their strengths and challenges in understanding the disease.

## 2. Lung Architecture and Physiological Structure

Lungs are paired, symmetrical organs that are part of the respiratory system. They are located within the chest in the two pleural cavities, laterally to the mediastinum.

The lung bud appears on the 26th day of gestation as an outgrowth of the primitive gut. It comprises undifferentiated epithelial cells that expand into the surrounding embryonic connective tissue or mesenchyme. The development of the functional components of the lungs, the alveoli, begins later: the alveolar phase starts almost at the end of gestation, around the 36th week, continuing into early childhood, around 18–24 months of age, resulting in the formation of a final number of alveoli estimated at around 200–300 million [11].

In an adult subject, lungs can be defined as sophisticated organs comprising semi-rigid conducting tubes branching and narrowing from the trachea, bronchi, and bronchioles, ultimately leading to highly vascularized sacs known as alveoli, where respiratory gases are exchanged.

These airways consist of various cell types, primarily from the embryonic neuroectoderm, mesoderm, and endoderm. Within the mature lung, different types of cells exist in specific proportions and locations, forming the structural elements crucial for ventilation. This intricate structure is shaped by continuous exposure to particles, pathogens, and toxicants through the secretion of protective mucus and surfactants, active mucociliary clearance, and an essential innate and acquired immune system [12].

The conducting airways in the lungs are primarily populated by four main types of epithelial cells: ciliated cells, goblet cells, and basal cells.

Ciliated cells facilitate mucus movement through synchronous cilia beating while goblet cells secrete mucus onto airway surfaces. Basal cells act as adult stem cells, predominantly lining larger airways [13].

**Figure 1:**
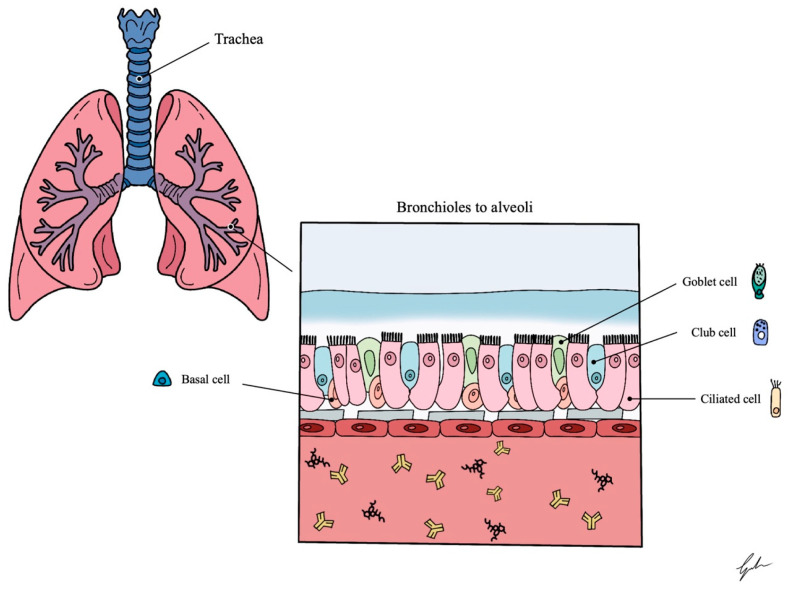
Schematic representation of the main cell lines that are present in the lung. It highlights the different compositions of the innermost epithelial compartment in the various portions of the respiratory tract. Adapted from Dichtl et al. [14].

Other sparse cell populations include club cells, which produce lubricating substances and antimicrobial peptides, and pulmonary neuroendocrine cells, which release proteins under physiological stimuli like hypoxia. Less common cell types include ionocytes and brush cells [12] (Figure 1).

The respiratory tract and lungs perform several vital functions in the body. They are essential in facilitating respiration, supporting pulmonary circulation, and contributing to the immune system.

Considering the complexity of this system, reliable physiological and pathological models are essential to enhancing our understanding of the mechanisms and pathomechanisms in the respiratory tract and lungs [14].

## 3. Tubercular Infection

TB, caused by the microorganism belonging to the *Mycobacterium tuberculosis complex* (MTBC), is a preventable infectious disease that typically enters the body through the respiratory tract, primarily targeting the lungs.

TB is transmitted via aerosol droplets with dimensions ranging from 1 to 5 µm. It affects 10 million people worldwide each year, and it is one of the infectious diseases with higher mortality rates. It is the world’s second leading cause of death from a single infectious agent [4,15].

The human-adapted Mtb is well known for its adaptability to the host immune system, allowing it to persist within the infected host. For this reason, TB eradication typically requires 6 to 9 months of multidrug chemotherapy, making it a complicated task [16].

Moreover, multidrug-resistant or rifampicin-resistant TB (MDR/RR-TB) presents a growing public health challenge in many regions, particularly in Eastern Europe, Russia, Asia, and Sub-Saharan Africa.

Considering what has been said before, immediate and rapid action is necessary to eliminate what is recognized as a global “TB epidemic” by 2030, a target endorsed by all United Nations (UN) Member States and the WHO [17].

**Figure 2:**
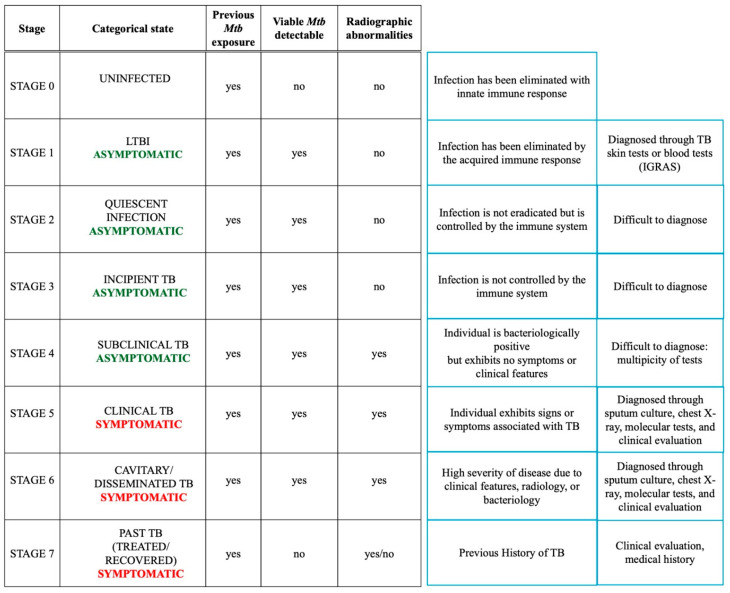
Schematic representation of the spectrum of TB disease.

Patients infected with Mtb can develop a wide array of symptoms and manifestations [18]. Traditionally, the Mtb infection has been described through a dichotomy: LTBI, which is asymptomatic, and ‘active’ TB disease (ATB), which is contagious and symptomatic. Recent advancements in understanding TB pathogenesis have highlighted that the step that takes us from Mtb infection to the tubercular disease is a more complex continuum identifying eight broad conceptual themes to categorize TB states, summarized in Figure 2 [19].

This distinction is particularly relevant because it clarifies that a symptom-driven approach alone may not reduce TB incidence. Comprehensive diagnostic strategies must address all phases of the disease, including subclinical TB, which remains difficult to diagnose. Developing reliable models is crucial to bridging the gaps in current procedures for diagnosing and treating this phase of TB [20].

The interaction between Mtb and macrophages is central to the progression of tuberculosis. These immune cells are the first to encounter Mtb during infection, attempting to engulf it through phagocytosis. However, Mtb has evolved mechanisms to evade destruction, including inhibiting phagosome maturation and preventing lysosome fusion. This allows Mtb to survive and replicate within macrophages, creating a protected intracellular environment.

Additionally, Mtb manipulates host immune responses by altering cytokine signaling and inhibiting apoptosis, supporting the formation of granulomas. Granulomas contain the infection but allow Mtb to persist in a latent state, complicating efforts to eliminate the disease by creating a reservoir of viable bacteria. Granulomas are composed of several resident cells (e.g., lung fibroblasts and epithelial cells), immune cells such as neutrophils, T-cells, and natural killer cells recruited by alveolar macrophages and by Mtb-infected alveolar macrophages [19].

After the infection is established, Mtb, like other bacteria, can go into dormancy.

In the dormant state, Mtb remains viable but inactive within infected cells or granulomas, avoiding immune detection and causing no symptoms. This dormant phase is key to the high proportion of individuals who remain asymptomatic and do not progress to active TB. Only about 5–10% of people infected with Mtb will develop active, symptomatic disease within the first five years, either shortly after infection or due to the reactivation of dormant bacteria later in life. When complete clearance does not occur, people develop what is called “latent infection”; this means that the host controls the pathogen’s replication and the individual remains asymptomatic but will be at risk of developing the disease in the active form during their lifetime [20,21].

Reactivation typically occurs when the immune system is compromised, such as in conditions like Human Immunodeficiency Virus (HIV) infection, malnutrition, or aging. This ability to switch between active and dormant states is central to the persistence and complexity of TB, making it challenging to diagnose and treat effectively [21].

Moreover, the Mtb capability to evade these host defenses by inhibiting phagolysosome formation, blocking phagosome acidification, expressing glycine-rich host proteins, and creating an electron-transparent zone (ETZ) that hinders the diffusion of lysosomal enzymes increases the chances for Mtb to persist in the host.

Moreover, recent studies have confirmed that many external stress stimuli can lead to different molecular responses that result in transcriptional, posttranscriptional, or allosteric controls in Mtb [20].

Given the factors discussed earlier, it is not difficult to understand why drug-resistant Mtb strains are evolving. Mtb bacilli can become resistant to anti-TB drugs by acquiring genetic mutations and utilizing various mechanisms to adapt under selective environmental pressure. A significant contributing factor is the phenomenon of heteroresistance, where subpopulations of Mtb within the same infection exhibit different levels of resistance to drugs, complicating treatment and eradication efforts [22].

The complex structure of granulomas poses additional challenges for drug penetration. The hypoxic and nutrient-deprived environment within granulomas can limit the efficacy of many anti-TB drugs, reducing their ability to reach therapeutic concentrations at the site of infection. Together, these factors make the treatment of drug-resistant TB particularly challenging, as both genetic mutations and pharmacokinetic barriers contribute to the bacteria’s survival and persistence [23].

### Pulmonary Microenvironment and Drug Resistance in Tuberculosis

The mechanisms underlying drug resistance in TB are intricately linked to the morphological and physiological characteristics of the lung, which significantly impact both diagnostic approaches and treatment efficacy. Radiographic techniques, such as chest X-rays, provide crucial insights into variations in lung morphology, including cavitations and fibrotic lesions, which delineate the pulmonary microenvironment that facilitates the persistence of Mtb [24,25]. These structural alterations, often accompanied by hypoxic and poorly vascularized regions, hinder drug penetration and contribute to the development of resistance. In parallel, drug-resistant Mtb strains undergo metabolic and molecular adaptations that enable survival in the hostile pulmonary environment, characterized by low oxygen availability, acidic pH, and increased oxidative stress [25]. Within alveolar macrophages, drug-tolerant Mtb employs specific survival strategies, including the evasion of host immune responses, further complicating therapeutic interventions [26].

Additionally, gradients of drug penetration within lung tissues, particularly in areas with suboptimal drug concentrations, are strongly associated with the emergence of acquired resistance [27]. These findings underline the pivotal role of the lung’s unique structural and physiological attributes in the persistence of drug-resistant TB. The complexity of these interactions highlights the necessity for developing more sophisticated models that accurately replicate the lung environment, enabling a more comprehensive understanding of the mechanisms of drug resistance. The establishment and validation of precise in vitro and in vivo models that simulate the heterogeneous conditions of the pulmonary microenvironment are critical for identifying novel therapeutic targets and optimizing drug efficacy. These models will provide valuable insights into the spatial and temporal dynamics of drug penetration, immune responses, and bacterial adaptation, thereby advancing our understanding of the processes that drive the emergence and persistence of drug-resistant TB.

## 4. How to Study the Pulmonary Infection: “In Vivo” Models

As we said before, the organs comprising the respiratory system often serve as a first barrier against exogenous substances and act as a filter for the inhaled air, primarily at the microbial level. Consequently, many infectious diseases affect the entire respiratory system. For this reason, deepening our understanding of the physiological and pathological mechanisms operating within the respiratory tract has become essential. To do this, it has been necessary to develop models.

“In vivo” models identify models used in experiments conducted in living organisms. Animal models have been crucial in uncovering the biochemical and physiological mechanisms underlying cancer and infectious diseases for decades. They are still essential for evaluating the safety and efficacy of new drugs or implantable devices. However, these models inadequately replicate the histological structure of the human lung epithelium and demonstrate shortcomings in mimicking physical characteristics and physiological processes such as cell-to-cell communication [4,28]. Moreover, the significant failure rates in clinical drug development and the challenges in translating preclinical animal data to human outcomes, alongside disparities between animal and human biology and human-specific pathogens, underscore the need for a paradigm shift towards advanced and validated human cell-based cultures [3].

### 4.1. Small Animal Models

**Figure 3:**
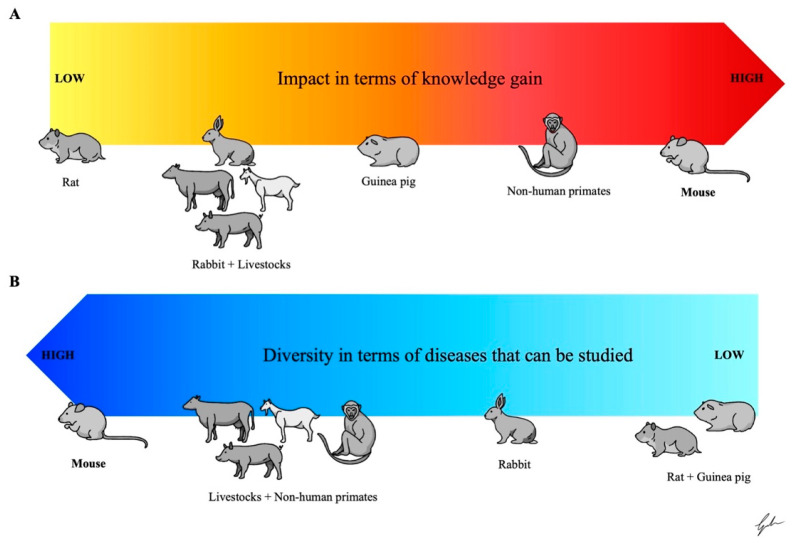
The figure represents a schematic overview of animal models for TB, reporting the diverse range of species used for studying these respiratory infections, from mice to non-human primates. Among these, non-human primates and mice have contributed the most to advancing knowledge, with non-human primates offering the highest translational value (**A**). On the other side, murine models are the most varied while guinea pigs, rabbits, hamsters, and livestock typically apply to only one of the two diseases (**B**). Adapted from Corleis et al. [4].

Throughout history, small animal models, such as those involving rodents and leporids, played a pivotal role as animal models for the study of pulmonary infections. Despite the limitations described above and better specified below, these models remain indispensable in preclinical TB research and have significantly contributed to the rapid progress in developing COVID-19 vaccines. Specifically, mouse models are the most widespread and used animal models, even if they only partially recapitulate the complexity of human disease. On the contrary, other rodent species like rats, hamsters, and guinea pigs (Figure 3) offer valuable insights into the pathophysiological aspects of respiratory infectious diseases that murine models may not fully capture.

#### 4.1.1. Mouse Model

Mice constitute the most used model for preclinical investigations due to their ease of handling, accessibility, affordability, and the extensive array of immunological and genetic tools available for research. Regarding Mtb infection, mice models are some of the first choices, considering their well-established nature in laboratory settings. However, while murine models offer numerous advantages, they also have limitations, particularly in fully capturing the complexities of TB infections [4,29].

The experimental murine TB model, achieved through aerosol exposure, has provided invaluable insights into the fate of the host following natural infection. It has meticulously delineated the intricate kinetics of the infectious process and facilitated the comprehensive integration of host and pathogen traits in experimental investigations.

However, mice do not fully replicate the TB pathology observed in humans. Critical features such as granuloma liquefaction, cavitation, and fibrosis remain absent in Mtb-infected mice. Nonetheless, this model offers a platform to examine immune responses to Mtb extensively. The similarity between pulmonary anatomy and immune mechanisms in mice and humans makes mice well suited to study immune dynamics within tissues and evaluate vaccine efficacy. Despite not being natural hosts for Mtb and generally exhibiting tolerance to the development of TB, mice have played a crucial role in elucidating specific disease mechanisms: the murine TB model has significantly advanced our understanding of immune responses to Mtb, especially in terms of granuloma formation, cellular interactions, and cytokine production. These insights have been instrumental in vaccine and drug development, particularly emphasizing immune responses in the lungs.

Overall, different aspects must be considered: host factors such as mouse genetics, age, sex, and immune status play pivotal roles in determining outcomes [30]. Additionally, variables including the route of infection, inoculum size, and bacterial characteristics such as Mtb lineages and virulence factors also significantly impact the progression of TB: exposure to different strains of Mtb in mice results in distinct outcomes, with some strains triggering heightened susceptibility and granulomatous lesions resembling human pathology. Various genetic modifications in Mtb or host mice, such as the deletion of virulence genes or immune-related genes, have been instrumental in elucidating the mechanisms underlying TB pathogenesis and host immune response.

Moreover, different strains of mice, including inbred, knock-out, collaborative cross (CC), and diversity outbred (DO) lines, have been employed to study TB. Inbred mice and knock-out lines have helped identify specific host factors essential for susceptibility to TB. In contrast, genetic diversity in CC and DO mice has facilitated the discovery of unbiased susceptibility or resistance traits [31].

In the end, it is essential to underline also the utility of transgenic mouse models in TB research. These models have provided insights into TB pathogenesis, including the role of immune cells, cytokines, and molecular pathways.

#### 4.1.2. Genetically Diverse Mouse Models in Tuberculosis Research

Genetically diverse mouse models are essential for studying infectious diseases as they mirror the genetic variability found in human populations. Utilizing outbred mouse strains, such as the CC or DO populations, researchers can investigate how genetic differences influence immune responses and disease susceptibility.

Researchers have observed substantial heterogeneity in bacterial load reduction across different combinations of mouse and Mtb strains, highlighting the impact of both host and pathogen genetic diversity on vaccine efficacy. This variability underscores the importance of incorporating genetically diverse models to understand disease mechanisms better and develop more effective, personalized treatments [32].

Additionally, it is important to underline how genetic diversity among mouse models influences TB outcomes. It emphasizes that different mouse strains exhibit varying immune responses to Mtb, leading to divergent disease outcomes. This variability underscores the necessity of selecting appropriate mouse models that accurately represent human genetic diversity to enhance the translational relevance of preclinical studies [33]. The findings highlight the significant impact of genetic factors on the response to treatment, reinforcing the importance of using genetically diverse models in tuberculosis research to assess the efficacy of new therapeutic agents [32].

#### 4.1.3. Rat Model

Rats, particularly Wistar rats, are often involved in the pharmaceutical industry for pharmacokinetic and toxicological studies. They are also commonly present in immunization studies because of their widespread availability, ease of handling, well-defined physiology, and capacity to yield larger samples compared to mice.

Rats generally exhibit susceptibility to Mtb infection and have been utilized to discern investigational compounds’ bacteriostatic or bactericidal properties [34]. Various rat strains, including American cotton rats, Lewis rats, Wistar rats, and Sprague–Dawley rats, develop granulomatous lesions that do not undergo liquefaction, thus failing to replicate human TB pathology fully [29]. Furthermore, rat models, particularly Wistar and American cotton rats, have been helpful for evaluating the bacteriostatic and bactericidal properties of investigational compounds. Rats develop granulomatous lesions that can be used to assess therapeutic interventions. Moreover, the rat model allows for studying immune responses to Mtb, especially concerning vaccine efficacy and the immune system’s ability to control bacterial proliferation.

#### 4.1.4. Guinea Pig Model

Guinea pigs are highly susceptible to TB: in his ground-breaking experiments, Robert Koch worked with guinea pigs and rabbits to demonstrate that pure cultures of Mtb cause the disease. They inhale mycobacteria and expel them through expectoration, akin to humans, making them suitable for transmission studies.

The course of TB infection in this type of animal varies based on the Mtb strain and initial dose, but invariably, animals succumb to Mtb infection. Following logarithmic lung growth, Mtb loads remain stable over many weeks. Toward the later stages of infection, Mtb bacteria can re-enter a logarithmic growth phase. Symptoms such as weight loss, labored breathing, and reduced activity are commonly observed, and this clinical observation aligns with histopathological findings of advanced disease. This later stage of infection is very similar to the humane endpoint. Granulomas in guinea pigs rarely exhibit liquefaction and cavitation [35]; they resemble primary lesions observed in humans more closely, making them helpful in assessing vaccine candidates and diagnostic tools. Therefore, guinea pigs are not suitable for studying mycobacterial latency.

Despite being a valuable model for TB diagnostics and compound testing, guinea pigs are not suitable for studying mycobacterial latency due to the limited ability of granulomas to exhibit liquefaction and cavitation. Recent advances, however, have seen the development of guinea-pig-specific monoclonal antibodies and molecular screening techniques, enhancing their utility in immunological and vaccine research [36].

#### 4.1.5. Rabbit Model

Rabbits constitute valuable models for studying the clinical features of Mtb infection [37]. The rabbit model closely reproduces post-primary TB, with animals developing cavities similar to human lesions involving bronchiole congestion, massive mycobacterial multiplication, and extensive necrotizing tissue destruction.

Moreover, also the outcome of Mtb exposure can be studied in rabbits, revealing that early innate inflammatory responses, the inoculum size, and bacillary aggregation facilitate progressive TB and the development of pathology rather than the establishment of LTBI: TB pathology in rabbits, particularly the occurrence of cavities, mirrors the critical stage of the disease that is most relevant for successful antibiotic treatment. Rabbits develop lung cavities, a crucial feature of post-primary TB in humans. This makes them suitable for studying disease progression, particularly in the context of bacterial multiplication and tissue necrosis. Moreover, they are essential for studying the biodistribution of new and established antimycobacterial compounds. However, like guinea pigs, rabbits lack immunological reagents, which limits vaccinology studies.

Additionally, the rabbit model is limited in replicating the clinical manifestations of TB. Furthermore, the genetic editing of rabbits is still in its early stages, and the high costs compared to rodent models restrict the use of the rabbit model to specific scientific inquiries [38,39]. Despite these challenges, rabbits remain part of a valuable model for studying the progression of TB and evaluating novel treatments.

### 4.2. Large Animal Models

Livestock species and non-human primates (NHPs) are natural hosts of the MTBC. Their similarity to humans regarding respiratory tract anatomy, lung structure (such as lobulation), and immune system organization and function makes them valuable models for studying respiratory diseases like TB and COVID-19. Their evolutionary proximity to humans provides additional advantages and unique insights into disease mechanisms.

#### 4.2.1. Non-Human Primate Model

Among NHPs, Rhesus Macaques (RMs), Cynomolgus Macaques (CMs), and African Green Monkeys are commonly used for research on Mtb infection [40].

Depending on factors such as the dosage of Mtb administered (ranging from 101 to 105 colony-forming units), the specific Mtb strain utilized, and the method of infection (intravenous, intratracheal, or aerosol), RMs and CMs can accurately replicate the entire spectrum of TB seen in humans, including acute infection, LTBI, and the reactivation of LTBI. Like humans, the granulomas observed in NHPs exhibit mature, adaptive structures characterized by necrotic cores surrounded by layers of macrophages and lymphocyte zones, including areas of immunocompromised microenvironments. NHP granulomas also feature tertiary lymphoid structures, which are crucial in antimycobacterial immunity and mirroring human lesions.

The NHP model is especially applicable for monitoring disease progression, including the formation of tertiary lymphoid structures, which play a critical role in antimycobacterial immunity. Furthermore, NHP models are pivotal for studying co-infection dynamics, such as MTB, HIV, or other respiratory pathogens. Their ability to replicate the full spectrum of human TB—from acute infection to latent TB infection (LTBI) and its reactivation—makes them indispensable for investigating immunopathological mechanisms and therapeutic interventions.

#### 4.2.2. Livestock Model

Large livestock species, including cattle, goats, and pigs, are natural hosts and serve as models for human TB. While Mtb is a human-adapted strain, other members of the MTBC, such as *M. orygis*, *M. caprae*, and *M. bovis*, are zoonotic pathogens, with livestock species, including cattle, goats, and pigs, serving as their main reservoirs [41], providing valuable models for investigating the pathology of zoonotic TB and studying potential diagnostic methods for extrapulmonary TB. These bacteria can infect humans and cause pathology like Mtb-driven disease, often resulting in extra-pulmonary manifestations. Interestingly, while Mtb can infect livestock, such as cattle, it usually does not induce comparable pathology. Under experimental conditions, cattle, goats, and pigs can eradicate Mtb. Therefore, livestock species can serve as models for human TB to investigate pathologies, such as that induced by M. bovis, and correlates of protection against Mtb.

In all species, macrophages and their precursors, such as monocytes, serve as the main intracellular niche for *M. bovis* or Mtb [42].

Large livestock species offer unique opportunities to investigate disease susceptibility and resistance in natural hosts and are valuable models for novel preclinical vaccine concepts.

While experimentation with cattle may be challenging due to their sizes and associated costs, goats offer a viable alternative. Pigs provide additional advantages such as their smaller size, lower costs, easier maintenance, and availability of immunological reagents [43]. Further studies in large livestock species are warranted to elucidate their potential as models for human TB.

Livestock species also allow for the investigation of disease resistance mechanisms and the development of diagnostic tools for extrapulmonary manifestations associated with zoonotic infections.

### 4.3. Limits and Perspectives of the “In Vivo” Models

Using animal models in infectious disease research offers invaluable insights into host responses and disease pathogenesis under controlled conditions. However, it is essential to recognize that no ideal model exists for studying diseases such as tubercular infection (Table 1).

Each animal model has benefits and limitations, and the purpose-oriented use of one or more models is essential for adequately addressing specific scientific questions and advancing interventions.

In conclusion, at the moment, the targeted and well-considered use of animal models remains indispensable for understanding infectious diseases and testing vaccines or therapeutics. However, it must be pointed out that it is essential to follow the 3R concepts to reduce, replace, and refine the usage of animals in experimental research. These ethical-driven approaches represent the foundation of animal experimentation around the world, and it is conceivable that in some cases, newer systems, such as three-dimensional cell culture or organoids, will continue to prove themselves to be able to replace some animal testing.

### 4.4. The 3Rs, “Replacement”, “Reduction”, and “Refinement”

The 3R principle—including Replacement, Reduction, and Refinement—refers to a widely accepted ethical framework for animal research. Its main aim is to minimize the use of animals in research by replacing them where possible, reducing the number of animals used, and refining procedures to lessen their suffering. This ethical concept has firm roots. Introduced in 1959 by British scientists W. M. S. Russell and R. L. Burch, it has now been incorporated into standard guidelines and regulations in many countries worldwide [44].

More in detail, even if the specific definition of the 3Rs might differ from one institution to another, it is generally accepted that “Replacement” deals with the possibility of alternative methods to minimize the use of animals as models in research [44,45]. “Reduction” deals with the scientific question that analyses the experimental protocol to understand whether it is possible to work with fewer animals than initially planned and explore whether parts of the problem can be investigated using non-animal testing methods. In the end, “Refinement” focuses on determining whether and how the suffering of laboratory animals can be minimized during an experiment or test.

Overall, as the directive 2010/63/EU specifies, the final goal of 3R is to completely replace animal research.

## 5. In “Vitro” Models

In vitro models are crucial in biomedical research. They offer a controlled environment for studying biological processes outside their natural contexts.

In vitro models are precious in studying Mtb infection because they allow exploring the interactions between the pathogen and the host immune system, which is not always achievable with in vivo models. This is particularly significant when considering the genetic variability of both the host (i.e., ethnic background) and the pathogen (different Mtb lineages). These factors can significantly influence infection dynamics and immune responses, making controlled in vitro systems indispensable for studying genetic and immunological intricacies.

In vitro systems allow researchers to investigate specific mechanisms and dynamic pathways that are challenging to study in animal models. Additionally, these models are indispensable for high-throughput drug screening as they are more cost-effective and less resource-intensive than in vivo studies. They are typically categorized into two-dimensional (2D) and three-dimensional (3D) cellular models, each providing unique insights into infection biology [14].

Two-dimensional culture techniques have provided standardized and controlled conditions for experiments with living biological specimens. However, they have limitations due to the lack of a physiological microenvironment. The main limitations are (i) differences in substrate stiffness, (ii) the absence of spatial cues, (iii) receptor topography, and (iv) differences in the concentration gradients of nutrients and gases. It must be underlined that substrate stiffness and morphology significantly influence differentiation, migration, and gene expression.

Three-dimensional models have emerged to overcome the limitations of 2D cultures. These models can potentially mimic physiological tissue behavior more accurately and at a lower cost than some animal models. By incorporating tissue complexity, 3D models provide more physiologically relevant environments for studying various biological processes, including matrix remodeling, cell–cell interactions, growth factor secretion, and gene regulation [14].

The development of biological 3D multi-tissue models requires a multidisciplinary approach, integrating engineering, chemistry, physics, mathematics, biology, and physiology. These models offer promising avenues for studying human organ responses to external stimuli, cytofunctionality analysis, and the design of implant substrates.

Additionally, they reduce the need for small animal models, aligning with the principles of the 3Rs (reduce, replace, refine) in animal experimentation.

In summary, 3D in vitro models represent a pivotal tool for evaluating essential biological processes in a physiological context, providing valuable insights into cell interactions and reducing the reliance on animal models in research.

## 6. Cell Culture of Human Airway Epithelial Cells: Focus on 3D Models and Their Application in the Study of the Tubercular Infection

**Figure 4:**
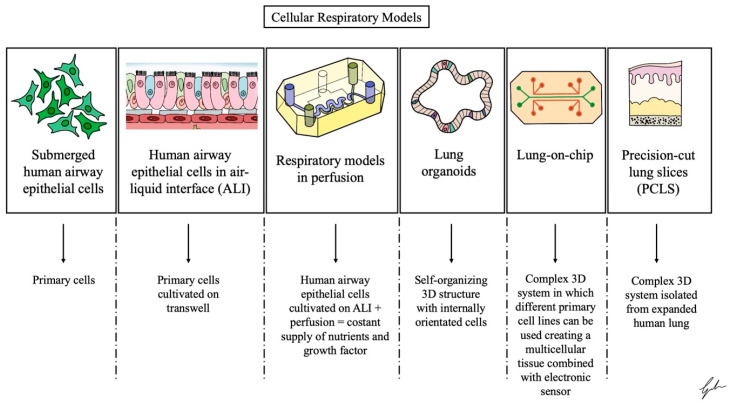
Schematic summarization of the most common 3D models used for reproducing the human airways. Adapted from Dichtl et al. [14].

Nasal, tracheal, bronchial, small airway, and alveolar epithelial cells can be cultured using various techniques, including submerged conditions, air–liquid interface (ALI) culture, or lung-on-a-chip systems or in organoid form (Figure 4) [46].

These cell culture models aim to mimic the characteristics of epithelial cells from their specific regions within the human respiratory tract or lung in vivo.

These models represent different types of epithelial cells, mirroring the diversity observed in the human lung. These include club, ciliated, basal, and goblet cells.

### 6.1. Submerged and ALI Culture

Human airway epithelial (HAE) cells derived from primary tissue offer a promising opportunity to study the human lung epithelium in various experimental setups. When HAE cells are cultured in a submerged environment, they are typically grown on plastic surfaces coated with extracellular matrix proteins like collagen or cellulose. This method yields primarily basal cells distributed throughout the airway tree but results in the loss of differentiated ciliated and secretory cells.

The air–liquid interface (ALI) culture technique has been developed to address these shortcomings and create a more physiologically relevant model. In an ALI culture, HAE cells are seeded onto microporous membranes coated with matrix proteins, exposing the apical side of the cell layer to air. In contrast, the basolateral side remains submerged in a nutrient medium. This setting enables HAE cells to undergo differentiation into a pseudostratified mucociliary epithelium encompassing various functional cell types, including basal, ciliated, and mucus-secreting goblet cells.

ALI-cultured HAE cells offer a closer representation of the in vivo lung environment. Additionally, an ALI culture presents the advantage of exposing the cells to pathogens or airborne substances, facilitating studies on host–pathogen interactions and respiratory toxicology [47].

### 6.2. Organoids

Organoids represent 3D structures that autonomously form tissue architectures resembling airways [48]. They mirror the critical characteristics of their respective organs. These respiratory cell aggregates originate from stem or progenitor cells sourced from adult or embryonic tissues or induced pluripotent stem cells (iPSCs) [49].

Typically, organoids derived from adult stem cells (ASCs) exhibit polarized, cystic structures primarily composed of epithelial cells. These structures are less complex than organoids derived from pluripotent stem cells (PSCs), which also contain non-epithelial cell types. The choice of stem cell source significantly influences the cellular composition of lung organoids. Human-PSC-derived organoids tend to display an alveolar phenotype, predominantly containing alveolar type I (AT1) and type II (AT2) cells.

Conversely, organoids obtained from adult tissue biopsies or bronchoalveolar lavage fluid present a broader range of cell types including basal, club, goblet, and ciliated cells [50].

While lung organoids contain fewer basal cells than HAE cells, they feature a higher abundance of club and ciliated cells.

Organoids are typically embedded in a matrix like Matrigel or cultured in 3D systems like the Celvivo system. These organoids maintain an internally oriented ciliated apical surface, which poses experimental challenges. Consequently, methods have been devised to reverse the epithelial polarity to expose the apical surface outward. Strategies to enhance proximal differentiation include inducing ciliary differentiation in alveolar organoids, resulting in an increased presence of ciliated, basal, and goblet cells while decreasing club cell populations.

Human organoid cultures can be differentiated on transwell inserts or as monolayers in submerged cultures [51].

Monolayer cultivation predominantly yields a distal signature with AT1 cells, suggesting a bias towards AT2 differentiation into AT1 cells. In contrast, ALI-cultured lung organoids comprise ciliated, goblet, and basal cells, forming a proximal pseudostratified mucociliary epithelium.

### 6.3. Lung-on-Chip Model

The “lung-on-chip” model utilizes complex 3D systems comprising primary cells, cell lines, PSCs, or ASCs on engineered microdevices. This model mimics the in vivo environment by incorporating multicellular tissues, which can be integrated with electronic sensors for real-time organ and tissue function monitoring.

Additional advantages include the combination of engineered microtissues with microfluidic devices, enabling the application of mechanical forces such as stretch and shear, fluid flow, biochemical cues, and electrical or optical signals [52].

### 6.4. Co-Culture with Immune Components

HAE or lung organoids cultured on a transwell—submerged or in ALI—can be supplemented with other cells such as immune cells.

The respiratory immune system is increasingly recognized for maintaining epithelial barrier integrity and lung homeostasis. Even epithelial disruption triggers the massive production of innate immune components, leading to inflammation or immune cell recruitment. Nonimmune epithelial cells can release innate immune responses like intracellular complements and cytokines. Therefore, incorporating immune cells or characterizing humoral immune components at infection sites is crucial for bridging the gap between in vitro models and the host situation.

During initial pathogen invasion, dendritic cells (DCs), macrophages, natural killer cells, and neutrophils synergistically promote airway inflammation, cytokine release, and the lysis of infected cells.

DCs, as immune sentinels, process antigens and transition from immature to mature cells, initiating adaptive immunity. Including these known modulators of respiratory barrier functions in in vitro models is essential for better mimicking the host environment. Immune cells are typically added on the basolateral side, interacting with epithelial cells through cell–cell contact or cytokine release.

Co-culture models, such as neutrophils with HAE cells, macrophages with HAE or SAE cells, or dendritic cells with HAE cells, are essential, especially for studying infection at entry sites [14,47].

The co-culturing of the airway epithelium with peripheral blood mononuclear cells allows the analysis of innate and adaptive immune subsets during infection over time. Incorporating immune cells like NK or CD8 T cells requires matching the HLA phenotype between immune and epithelial cells [53,54,55].

### 6.5. Respiratory Models in Perfusion and Precision-Cut Lung Slices

Microphysiological systems, like organ-on-chip and interconnected 3D tissue constructs, provide more relevant alternatives to traditional cultures or animal studies, especially for studying human–pathogen interactions at barrier sites. In vitro microfluidic models for the respiratory tract, including at the alveolar level, mimic respiration and vascularization, offering a more accurate representation. These systems continuously evolve to incorporate the respiratory microenvironment’s physical, mechanical, and organizational features [56].

Perfused conditions in microfluidic models significantly enhance ciliogenesis and mucus production compared to static cultures. This is attributed to the increased nutrient supply and enhanced cell communication.

Precision-cut lung slices from human lungs offer another ex vivo model maintaining microarchitecture and function, enabling mechanistic studies.

However, their viability is typically limited to 7–10 days, with changes in tissue architecture over time posing some limitations. Despite this, precision-cut lung slices remain valuable for controlled lung physiology and pathology investigations.

The table below (Table 2) reports the main advantages and disadvantages of 2D and 3D in vitro models, highlighting their applications and limitations in respiratory research and beyond. Special emphasis is given to advanced 3D models, such as ALI cultures, organoids, lung-on-chip systems, and co-culture techniques, which offer unique insights into biological processes.

## 7. In Vitro Granuloma Models of Tuberculosis

Despite extensive research, many fundamental aspects of TB pathogenesis remain elusive. The intricate interplay between the host and the pathogen, particularly within multicellular tissue granulomas, is a crucial challenge.

**Figure 5:**
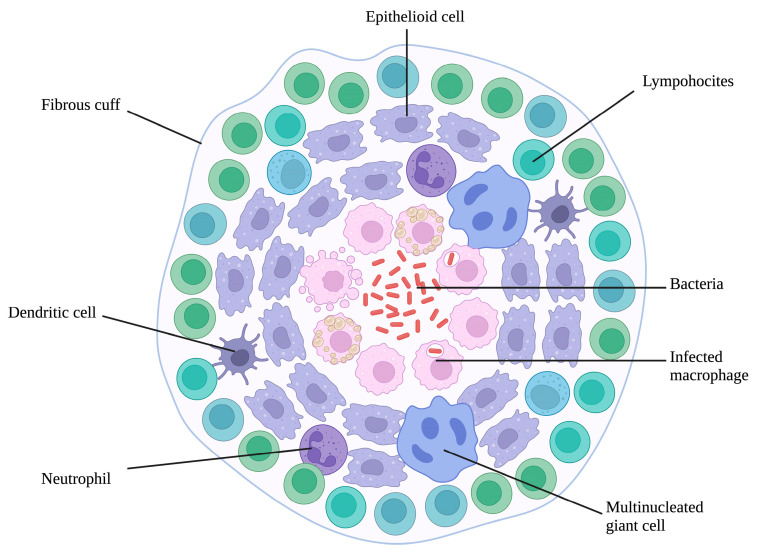
Schematic representation of the main cellular component of a granuloma. Created in BioRender. https://BioRender.com/m59m113 (accessed on 18 December 2024).

Granulomas are complex structures composed of various cell types. At the core, they are predominantly mature macrophages surrounded by T cells, B cells, and fibroblasts. They are formed in response to persistent stimuli (Figure 5).

While granulomas have been observed for centuries, the mechanisms governing their dynamics, behavior, and maintenance are now being elucidated [57].

Contrary to traditional views, in which granulomas have always been represented as ways of limiting infection, recent findings from the zebrafish model suggest that in *Mtb* infection, they may promote mycobacterial growth and spread.

For this reason, understanding the molecular basis of macrophage reprogramming and specific cellular events within tuberculosis granulomas, like multinucleate giant cell formation and central necrotic caseation, still needs to be completed.

### 7.1. Cell Culture Systems of TB Granulomas

Humanized organoids have emerged as powerful tools for studying infectious diseases. They offer a more physiologically relevant platform that closely mimics human tissue. These 3D models, derived from human stem cells, provide insights into host–pathogen interactions, immune responses, and disease progression in a genetically accurate context. Humanized organoids can be engineered to replicate specific genetic traits or disease conditions, making them particularly useful in tuberculosis research, where understanding the intricate dynamics between Mtb and human immune cells is critical.

Recent advances in using lung-specific organoids have been pivotal in exploring how various factors, such as cigarette smoke exposure or genetic predispositions, affect tuberculosis outcomes. By incorporating elements like macrophages, epithelial cells, and other immune components, researchers can replicate the pulmonary microenvironment more effectively than traditional 2D cell culture models. This setup allows for a more comprehensive study of immune responses, such as inflammation, cytokine production, and cell-mediated immunity, which are crucial for understanding tuberculosis pathogenesis. Furthermore, 3D human organoids offer a new dimension for exploring the molecular basis of infectious diseases, making them an essential tool in studying the impact of environmental factors like nicotine on Mtb infection. These advanced models may provide insights into oxidative stress and other disease mechanisms, supporting the development of targeted therapeutic strategies for tuberculosis [58].

#### 7.1.1. Collagen Matrix Model of *M. tuberculosis* Dormancy

Kapoor and colleagues [53] developed a 3D model that employs human peripheral blood mononuclear cells (PBMCs) within an extracellular matrix to recreate spatially and temporally accurate three-dimensional structures and microscopic granulomatous aggregates in response to virulent *Mtb*. This model effectively mimics key features of human tuberculosis, such as multinucleated giant cell formation, changes in T cell populations, macrophage activation status, and increased cytokine and chemokine secretion upon *Mtb* exposure.

A crucial aspect of this model is its ability to simulate *Mtb* latency and reactivation under immune suppression induced by anti-tumor necrosis factor-alpha (anti-TNF-α) treatment. Thus, it offers insights into host–pathogen interactions during latency and reactivation, showcasing fundamental latency characteristics, including non-replicating states, rifampicin resistance, the loss of acid-fastness, and lipid body accumulation.

Despite its strengths, the model has limitations such as low throughput, technical challenges in adding cells over time, and the need for collagenase to release cells from the matrix for analysis. Nonetheless, its potential to advance our understanding of *Mtb* latency and reactivation and identify therapeutic interventions remains promising [59].

#### 7.1.2. Multicellular Lung Tissue Model

Lerm and colleagues introduced *Mtb* into a human in vitro lung tissue model to investigate early granuloma formation [60] This model involves a collagen matrix supported by a filter membrane acting as a scaffold for a human fibroblast cell line, which grows and differentiates before the addition of primary macrophages/monocytes and a human epithelial cell line.

Once tissue formation occurs, the apical side is exposed to air, stimulating mucus secretion by the epithelial cells. This organotypic mucosa closely resembles lung tissue both anatomically and functionally.

Studies in zebrafish and human lung tissue models suggest that *Mtb* virulence factors are involved in the induction of granuloma formation. For instance, host matrix metalloproteinases (MMPs) induced by mycobacterial virulence factors are known to promote granuloma formation [61]. The inhibition of MMPs has been shown to reduce both granuloma pathology and bacterial load in the human lung tissue model, aligning with reports advocating for MMP inhibition in mouse models of tuberculosis.

This model’s limitations include (i) challenges in introducing other immune cells, such as lymphocytes or neutrophils, and (ii) adapting it for high-throughput assays. Nevertheless, the model can potentially study host–pathogen interaction and secondary assays for assessing novel tuberculosis drugs.

#### 7.1.3. Granuloma Model to Assess the Impact of the Human Immune Response

Guirado et al. developed a granuloma model using human PBMCs and autologous serum to mimic the immune environment typical of Mtb infection [8]. Indeed, this innovative system allows the study of early granuloma formation. The model was developed to study the immunopathogenesis of individuals with LTBI. The authors demonstrated that LTBI subjects exhibit significant differences in bacterial survival, immune cell activity, cytokine production, and lipid body accumulation, highlighting variations in immune responses and their effects on granuloma formation. Additionally, it highlights a unique bacterial transcriptional signature influenced by the host’s immune status. The model’s ability to simulate these interactions demonstrates its potential for personalized medicine and advances in TB biomarker and treatment research.

Limitations of the initial model, such as the absence of fibroblasts and extracellular matrix components, have been addressed, influencing the kinetics and stability of granuloma formation. Additionally, the model lacked a continuous influx of mononuclear phagocytes needed for sustained dynamic structures, leading to the introduction of HIV into the model to explore its impact on granuloma formation and dissolution timing.

This model allows comparative analysis with other granulomatous diseases and can be adapted for the high-throughput screening of potential therapeutic compounds. The continuous refinement and development of this system offer promise for advancing the understanding of granuloma biology, exploring therapeutic interventions, and studying the effects of comorbidities.

#### 7.1.4. Bioelectrospray 3D Model

Workman et al. developed an innovative system based on cell encapsulation within microspheres, achieved through alginate cross-linking in a calcium chloride bath [62]. This technique produces highly customizable microspheres with a matrix and cellular composition conducive to manageable experimentation. Cells can be quickly released from the spheres for downstream analysis by dissolving them in solutions like EDTA or sodium citrate.

This system has been utilized to explore various aspects of the host–pathogen interaction and has several translational applications. Initially, comparisons between spheres with or without collagen revealed the regulatory role of the extracellular matrix in the host–pathogen interaction, consistent with findings from transgenic mouse studies. Subsequently, the group investigated different dimensions of the host immune response, including studies on spheres exposed to various cytokines or featuring enhanced *Mtb*-responsive T cells [63].

Integrating diverse cell types into multiple spheres and examining their effects over extended periods (up to 21 days) offers an effective tool for discerning protective versus pathological immune responses. Notably, *Mtb* exhibits sensitivity to pyrazinamide when cultured in three-dimensional microspheres, replicating stress conditions encountered in vivo. Moreover, due to the confinement of cells and bacteria within the microspheres, the model lends itself to microfluidic pharmacokinetic modeling. In this setup, increasing concentrations of rifampicin have been demonstrated to accelerate Mtb killing.

Additionally, the model has been used to explore the effects of MMP inhibition with doxycycline in limiting tuberculosis-driven immunopathology.

Like other systems, this model faces challenges, including difficulties incorporating additional immune cells after encapsulation within the microspheres. Further refinements have addressed these limitations by introducing a dual encapsulation system to mimic better granuloma features such as a caseous central core and an oxygen gradient from the periphery to a hypoxic center (casuem). These advancements could also facilitate cellular intake, enabling the model to simulate immune cell recruitment to granulomas more effectively, thereby enhancing its utility for studying tuberculosis immunopathogenesis.

#### 7.1.5. Lessons from Animal Models

The complexity of chronic host–pathogen interactions in human TB necessitates using animal models alongside in vitro systems. While in vitro models with human cells offer valuable insights, they lack the full complexity observed in human disease.

On the other hand, animal models provide a more comprehensive understanding of these interactions, with findings from such models informing the development of human systems.

A classically described granuloma in tuberculosis has a characteristic architecture, typically consisting of a caseous center surrounded by layers of myeloid and lymphocytic cells. These lesions may eventually become necrotic or fibrotic, leading to mineralization or cavitation. Signaling cascades involving myeloid cells and interferon-gamma from T cells aim to eliminate *Mtb* within the lesion. Still, sterilization is rarely achieved due to the complex granuloma environment, which promotes and inhibits bacterial killing. As we saw before, traditional mouse models do not fully replicate the specialized architecture of human lung TB granulomas. However, recent developments have allowed for generating necrotic or fibrotic lesions in mice. Other animal models, such as guinea pigs, rabbits, and macaques, develop granulomas more akin to those observed in humans, including necrotic and organized structures, with hypoxia particularly prominent in necrotic regions. Under specific conditions, rabbits have been used to study cavitary lesions, further highlighting the relevance of animal models in understanding tuberculosis pathogenesis.

These animal models provide crucial insights into the mechanisms within lung granulomas that drive the balance of *Mtb* killing and survival.

Conversely, 3D models are particularly useful in studying Mtb because they closely replicate granulomas’ structural and functional complexity, which is central to TB pathology. These models provide a controlled environment that allows researchers to investigate the interaction between Mtb and host immune cells.

Crucially, 3D models enable the assessment of the genetic background of both the host (e.g., ethnic variability) and the pathogen (e.g., Mtb lineages). This is important as both factors significantly influence infection outcomes and immune responses. Unlike traditional 2D or animal models, 3D systems can integrate human-relevant genetic and immunological diversity, facilitating more precise studies on how these variables shape granuloma formation, pathogen persistence, and immune evasion mechanisms.

Additionally, 3D systems can mimic oxygen gradients, nutrient limitations, and the architecture of granulomas more effectively than other models, making them indispensable tools for drug testing and personalized medicine research.

## 8. Conclusions and Final Perspectives

This review has provided a comprehensive overview of models used to study TB infection. It has focused on the interactions between TB and the host immune system, particularly within the context of granuloma formation. The review has highlighted the limitations of traditional 2D and animal models and underscored the growing relevance of 3D models for investigating TB pathogenesis and treatment strategies.

This review has aimed to emphasize the ability of 3D models to integrate the genetic diversity of both host (e.g., ethnic backgrounds) and pathogen (e.g., Mtb lineages). This aspect is crucial as genetic variability plays a key role in infection dynamics, immune responses, and disease progression. Our review has also outlined how 3D systems replicate essential features of granulomas, such as oxygen gradients, nutrient limitations, and tissue architecture, offering a more accurate simulation of the TB microenvironment. It has also pointed to the utility of 3D models in drug screening and personalized medicine by providing a cost-effective and ethically favorable alternative to animal models.

Moreover, understanding protective innate or adaptive immune responses characterized in these models can inform the development of new vaccination strategies and their efficacy in clinical settings.

The main challenge in designing new 3D models is accurately mimicking the physiological pathological microenvironment to reflect the pathogenesis. Developing increasingly complex models without a clear focus on answering specific pathophysiological questions could be wasteful. Thus, the development of granuloma systems should be closely correlated with observations in patients and animal models to ensure relevance to human disease.

A multidisciplinary approach, bringing together experts in advanced cell culture modeling, bioengineering, mathematical modeling, and in vivo experimentation, is essential to bridge the requirements of complexity, tractability, and throughput. Ultimately, a comprehensive understanding of tuberculosis pathogenesis is crucial for controlling the disease, and the further advancement of cell culture models and related technologies can play a central role in connecting in vivo experimentation with clinical studies.

## Figures and Tables

**Table 1:** Advantages and limits of the different in vivo models.

Model	Advantages	Limits	Applicability
Mouse	detailed immunological insights similarity to human anatomy and immune mechanisms genetic diversity feasibility of gene editing	imperfect disease model (lack of granuloma liquefaction, cavitation, and fibrosis) inadequacy for transmission studies	immunological research: immune dynamics within tissues and vaccine efficacy host susceptibility or resistance trait
Rat	broad availability easy handling defined physiology defined physiology potential to obtain larger samples compared to mice	inadequate mirroring of human TB pathology (the observed lesions do not fully replicate the liquefying lesions seen in humans) limitations in understanding TB pathophysiology	
Guinea Pig	susceptibility to TB and transmission mode entirely similar to humans revealing disease mechanisms human-like clinical presentation: guinea pigs develop granulomatous lesions similar to primary lesions in humans	scarcity of immunological tools lack of genetically modified strains limited understanding of complex interactions	vaccine efficacy transmission studies evaluation of diagnostic skin tests and antimycobacterial compounds
Rabbit	rabbit model closely reproduces post-primary TB, including cavity formation	lack of immunological reagents limitations in clinical manifestation infancy of genetic editing high costs	clinical features of TB: in particular the post-primary phase study of the antimycobacterial compounds
Non-human primates	reflection of human TB spectrum (granuloma formation) structural similarities	high housing costs ethical concerns shortage of animals experimental variability	monitoring disease progression: focusing on the role of tertiary lymphoid structures co-infection studies
Livestock animals	natural infection granuloma formation immune responses	high housing costs and difficult logistics ethical concerns experimental variability translation to humans: risk	transmission studies

**Table 2:** Summary of the advantages and disadvantages of 2D and 3D in vitro models including specific subcategories of 3D systems. The table highlights their relevance in mimicking physiological conditions and studying complex biological interactions.

Model	Advantages	Limits
2D Models	provide standardized and controlled conditions for experiments. simpler and more cost-effective compared to 3D or in vivo models. useful for high-throughput drug screening.	lack a physiological microenvironment. differences in substrate stiffness compared to in vivo tissues. absence of spatial cues and receptor topography. lack of nutrient and gas concentration gradients.
3D Models (General)	better mimic physiological tissue behavior compared to 2D models. provide a more physiologically relevant environment for studying complex processes (e.g., cell–cell interactions, gene regulation, tissue remodeling). reduce reliance on animal models, aligning with the 3R principles (Reduction, Replacement, Refinement). allow integration of immune components to better mimic the host microenvironment.	require multidisciplinary expertise for their development (e.g., engineering, biology, chemistry). more complex to set up and maintain compared to 2D models, making some experiments more challenging. advanced technologies (e.g., lung-on-chip) can be expensive and require specialized infrastructure.
ALI Culture	mimics the in vivo lung environment, allowing differentiation into a pseudostratified mucociliary epithelium. facilitates pathogen or airborne substance exposure studies.	limited primarily to epithelial cell studies without additional complexity unless combined with immune cells or other co-culture systems. requires specific substrates and culture conditions to maintain differentiation.
Organoids	resemble airway architecture, offering a model for studying specific regions of the respiratory tract. derived from diverse cell sources (e.g., adult tissues, iPSCs), allowing for specific cell-type representation. enable the study of differentiation and specific cellular functions (e.g., AT1 and AT2 cells, ciliated and goblet cells).	challenges in handling due to internal ciliated apical surfaces. less complex organoids (e.g., from adult tissues) primarily contain epithelial cells, lacking other cell types present in vivo. require advanced techniques to reverse polarity or enhance differentiation for certain applications.
Lung-on-chip	simulates in vivo environment with multicellular tissue layers and microfluidic devices, enabling real-time monitoring of organ and tissue function. incorporates mechanical forces (e.g., stretch, shear), fluid flow, and biochemical cues for studying dynamic processes. useful for studying respiration, vascularization, and infection dynamics at the alveolar level.	technically demanding and cost-intensive. requires specialized engineering knowledge and equipment for operation. limited adoption due to high complexity and cost compared to simpler 3D models.
Co-culture models	integrate immune components (e.g., dendritic cells, macrophages, T cells) with epithelial cells for a comprehensive study of host–pathogen interactions. allow the analysis of innate and adaptive immune responses, including cytokine release and immune cell recruitment.	require careful HLA matching between immune and epithelial cells for advanced co-culture studies. complexity increases with the addition of multiple cell types, making reproducibility and scalability challenging.
Precision Lung Slices	maintain lung microarchitecture and function, enabling mechanistic studies in an ex vivo setup. provide a controlled platform for studying lung physiology and pathology, including infection dynamics.	limited viability (7–10 days) with gradual loss of tissue architecture over time. small sample size and variability between donors can limit the reproducibility and generalizability of results.
